# Problems with sickness certification tasks: experiences from physicians in different clinical settings. A cross-sectional nationwide study in Sweden

**DOI:** 10.1186/s12913-015-0937-6

**Published:** 2015-08-12

**Authors:** Therese Ljungquist, Elin Hinas, Gunnar H. Nilsson, Catharina Gustavsson, Britt Arrelöv, Kristina Alexanderson

**Affiliations:** Department of Clinical Neuroscience, Division of Insurance Medicine, Karolinska Institutet, SE-171 77 Stockholm, Sweden; Stockholm County Council, Stockholm, Sweden; Department of Neurobiology, Care Sciences and Society, Centre for Family Medicine, Karolinska Institutet, Stockholm, Sweden

**Keywords:** Sick leave, Sickness certification, Physician, General practitioner, Primary health care, Insurance medicine

## Abstract

**Background:**

Many physicians find sickness certification of patients problematic. The aims were to explore problems that physicians in different clinical settings experience with sickness certification tasks in general and with assessment of function, work capacity, and need for sick leave, as well as handling of sick-leave spells of different durations.

**Methods:**

Data from a questionnaire sent to 33 144 physicians aged <68 years, living and working in Sweden in 2012 were analysed. The response rate was 57.6 %. The study group comprised the 12 933 responders who had sickness certification tasks. Frequencies and odds ratios with 95 % confidence intervals were calculated for questions concerning how problematic the physicians experienced different assessments related to patients’ function, work capacity, and need for sick leave, as well as handling sick-leave spells of different durations.

**Results:**

There were large differences between clinical settings regarding how often and to what extent sickness certification consultations were perceived as problematic. Physicians working in primary health care (PHC) had the highest proportions experiencing sickness certification consultations as problematic at least once a week (49.5 %) and as very or fairly problematic (56.6 %), followed by physicians working in psychiatry, pain management, or orthopaedics. More than half of the responders found it very or fairly problematic to assess patients’ work capacity (57.8 %), to make a long-term prognosis about patients’ future work capacity (55.7 %), and to handle long-term or very long-term sickness certifications (51.9 % and 51.8 %). The proportions were highest among physicians working in PHC, rheumatology, neurology, or psychiatry.

**Conclusions:**

The rates of physicians finding sickness certification task problematic varied much with clinical setting, and were highest among physicians in PHC. More knowledge is needed about the work conditions and prerequisites for optimal handling of sickness certification in different clinical settings.

## Background

In many Western countries, physicians from different types of specialties are involved in sickness certification of patients [1–11]. Also in most Western countries, the consultations where sickness certification is considered involve several different tasks for the physician to handle [12], specified in Sweden as follows [3, 13].

- determine if the patient has a disease or injury, that is, establish diagnoses,
- determine if and how the disease or injury impairs the patient’s function to the extent that work capacity is also impaired - in relation to the demands of the patient’s work,
- together with the patient consider the advantages and disadvantages of being sickness absent,
- determine the degree (full- or part-time) and duration of sick leave, and what actions that need to be taken during the sick-leave period in terms of investigations, treatments, rehabilitation, life style interventions, etcetera,
- determine the need for contact or collaboration with others within and outside of the health care system, e.g., a physiotherapist or employer – and establish such contacts, if needed
- issue a certificate that provides sufficient information to those who decide whether the patient is entitled to sickness benefits, and
- document relevant decisions, measures, and strategies planned.

Physicians seldom have enough training in such tasks [14–21]. Nevertheless, the way those tasks are handled has great influence on the life situation of patients and their families, and also has economic impact for employers, insurances, and nations. In Sweden, all physicians can write sickness certificates, and such are needed after the 7^th^ day of a sick-leave spell. All people with income from work or unemployment benefits are covered by the public sick-leave benefit insurance [22]. Interventions have been conducted in Sweden as well as in other countries to increase the competence of physicians regarding sickness certification tasks and thereby increase the quality of how they are handled [6, 14, 20, 23–32].

Systematic reviews of studies of physicians’ sickness certification practices have established that many physicians find sickness certification tasks problematic [3, 33, 34]. According to a previous study, based on a cross-sectional survey to all the physicians living and working in Sweden in 2008 [4], the two tasks that most physicians found problematic were assessing patients’ work capacity and providing a prognosis regarding the duration of work incapacity. The highest proportions finding these tasks problematic were found among physicians working in primary health care (PHC), rheumatology, psychiatry, neurology, or orthopaedics. In 2012 we sent a similar questionnaire to physicians living and working in Sweden. The aims of the present study were to explore how problematic physicians in different clinical settings experience sickness certification tasks in general as well as regarding specific issues related to assessment of function, work capacity, and need for sick leave.

## Methods

A cross-sectional study was conducted, based on data from a questionnaire sent to the 33 144 physicians aged <68 years, living and working in Sweden in October 2012, with the exception of board-certified specialists working in clinical settings where sickness certification seldom is handled, e.g., geriatrics, child healthcare, laboratory clinics, ophthalmology, and ear, nose and throat clinics [4, 35]. The included physicians were identified by Cegedim AB, a company that manages a register of all physicians in Sweden. The register includes information about age, sex, and specialist status provided by the National Board of Health and Welfare.

The comprehensive questionnaire, with 163 items about physicians’ work with sickness certification, was based on previous questionnaires [4, 35] and somewhat revised. The survey was administered by Statistics Sweden, who mailed the questionnaire to the physicians’ home addresses, in order to avoid interaction with colleagues when answering the questions. The physicians were informed about the purpose of the survey and that participation was optional and anonymous. Their informed consent for participation in the study was obtained through them answering the questionnaire. A prepaid envelope was enclosed, and alternatively, it was possible to answer through a web-based version, which 19 % did. Three reminders were sent to non-responders. Statistics Sweden conducted analyses of non-responders, based on available data. There was no information on numbers working in different clinical setting why non-response analyses related to that was not possible. Anonymous data for the responders were, thereafter, sent to the research group.

Answers to the following questions were included in the analyses:

1. At what type of clinic/practice do you mainly work?
2. How often in your daily clinical work do you have consultations including consideration of sickness certification (More than 10 times a week/6-10 times a week/1-5 times a week/About once a month/A few times a year/Never or almost never)?
3. How often in your clinical work do you find it problematic to handle sickness certification (More than 10 times a week/6-10 times a week/1-5 times a week/About once a month/A few times a year/Never or almost never)?
4. How problematic do you generally find it to handle sickness certification of patients (Very/Fairly/Somewhat/Not at all)?
5. How problematic do you generally find it to …, followed by ten different specific questions related to assessment of function, work capacity, need for sick-leave, as well as handling of sick-leave spells of different durations, listed in Table 3 (Very/Fairly/Somewhat/Not at all, and for the three questions concerning sick-leave spells of different duration, also Not applicable)?

Also, information on age, sex, and specialist status was used in the analyses.

The internal attrition rate on specific questions was on average 3.5 %.

### Statistical analyses

Descriptive statistics were used to describe the study group and answers to the questions listed in Table 2 and 3. Chi^2^ tests were used to analyse differences in sex, age, and specialist status on questions concerning frequencies of having sickness certification consultations, of finding such consultations problematic, and how problematic the handling of sickness certification was perceived (questions 2–4 above).

Logistic regressions were used to calculate odds ratios (OR) with 95 % confidence intervals (CI) for the questions concerning how problematic different assessments related to patients’ function, work capacity, and sick leave were experienced (question 5, as described above), using physicians working in internal medicine as reference group. That group was chosen as reference as they constituted a large group whose answers were close to the average for all physicians regarding the questions analysed. The ORs were adjusted for age (continuous variable) at the time for answering the questionnaire, as age was found to be a confounder, as opposed to other possible confounders tested, e.g., sex and specialist status.

SPSS statistics version 20 was used for the analyses.

The study was approved by the Regional Ethical Review Board of Stockholm.

## Results

The response rate was 57.6 % (19 107 physicians) and was somewhat higher among women and physicians in the older age group (Table 1). Responders who answered that they had not been working as a physician during the last 12 months, or that they mainly worked in another country, were not to fill in the rest of the questionnaire (*n* = 1185). In this study, the physicians who had consultations concerning sickness certification at least a few times a year were included (*n* = 12 933). Physicians who had not answered the question about clinical setting (*n* = 62) and those who answered ‘None’ (*n* = 21) were included in the clinical setting that best matched their specialist status/training. Sixteen types of clinical settings are presented in the results, physicians in primary health care (PHC) by far constitute the largest group (Table 1).

**Table 1:** Study population characteristics, response rate, number, and proportion of physicians having sickness certification consultations, stratified by clinical settings. Percentages represent % of the numbers in the previous column

					Among sick-listing physicians		
	Study population	Responders and response rate	Working as physicians in Sweden	Sick-listing physicians	Women	Specialists	Mean age
	n	n (%)	n (%)	n (%)	(%)	(%)	(years)
All	33144	19107 (57.6)	17922 (93.8)	12933 (72.2)	49.3	70.1	47
Men	17952	9873 (55.0)	9240 (93.6)	6563 (71.0)	-	76.5	49
Women	15192	9234 (60.8)	8682 (94.0)	6370 (73.4)	-	63.6	45
24–39 years	9966	5676 (57.0)	5410 (95.3)	4151 (76.7)	59.5	24.2	33
40–54 years	11921	6293 (52.8)	6031 (95.8)	4350 (72.1)	50.3	85.5	47
55–67 years	11257	7138 (63.4)	6481 (90.8)	4432 (68.4)	38.7	98.0	60
Non-specialist	9704	5422 (55.9)	5010 (92.4)	3866 (77.2)	60.0	-	35
Specialist	23440	13685 (58.4)	12912 (94.4)	9067 (70.2)	44.7	-	52
*Type of clinic*							
Primary health care			4183	4088 (97.7)	52.1	66.8	48
Internal medicine			1874	1756 (93.7)	45.7	65.7	44
Surgery			1515	1333 (88.0)	34.3	67.4	45
Psychiatry			1084	993 (91.6)	55.0	71.1	49
Gynaecology/Obstetrics			997	877 (88.0)	72.3	75.5	47
Orthopaedics			909	864 (95.0)	22.3	72.6	46
Oncology			361	346 (95.8)	63.0	75.1	47
Occupational health service			351	336 (95.7)	46.4	95.5	58
Infectious diseases			336	322 (95.8)	50.0	70.5	44
Neurology			267	252 (94.4)	46.8	71.0	45
Dermatology			244	176 (72.1)	69.9	80.7	49
Rheumatology			186	182 (97.8)	62.1	81.9	49
Rehabilitation			138	130 (94.2)	61.5	75.4	50
Pain management			86	65 (75.6)	33.8	96.9	54
Other			4487	1147 (25.6)	50.8	69.3	47
Administration			904	66 (7.3)	51.5	83.3	53

**Fig. 1:**
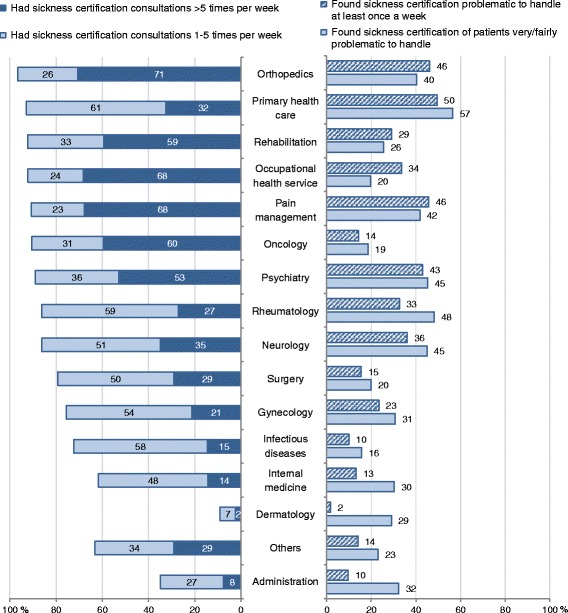
Rate of physicians in different clinical settings who had sickness certification consultations >5 times a week/1-5 times a week (to the left), and rate of physicians who found sickness certification problematic ≥ once a week or very/fairly problematic to handle (to the right)

In Fig. 1 we show the frequencies of sickness certification consultations in different clinical settings to the left, and both frequency and extent of finding such tasks problematic to the right. The proportion of physicians who stated having sickness certification consultations at least six times a week was highest among physicians working in orthopaedics (70.7 %), occupational health service (68.7 %), pain management (67.7 %), oncology (59.6 %), rehabilitation (59.2 %), or psychiatry (52.8 %). The proportions who experienced sickness certification consultations as problematic at least once weekly (i.e., frequency) and as very or fairly problematic (i.e., extent) were highest among physicians working in PHC (49.5 and 56.6 %, respectively), followed by physicians working in psychiatry, pain management, or orthopaedics (Fig. 1). Physicians working in oncology had sickness certification consultations frequently, however, a relatively low proportion of them found these tasks problematic. A similar pattern, though not as prominent, was found among physicians working in surgery.

In Table 2, frequencies of sickness certification consultations and of finding them problematic as well as to what extent the handling of sickness certification was perceived as problematic are presented by sex, age, and specialist status. There were significant differences (*p* < 0.001) in answers between men and women, all age groups, and specialist versus non-specialists for all questions. The proportion having such consultations at least six times per week, and that found them problematic as often, was higher among men compared to among women, whereas the proportion finding sickness certification very or fairly problematic to handle was higher among women. The proportion having such consultations at least six times per week was higher among specialists, whereas the non-specialists were more likely to find it problematic to handle sickness certifications.

**Table 2:** Proportion of physicians having sickness certifications and of finding them problematic. Stratified by sex, age, and specialist status (*n* = 12,933)

	How often do you have consultations including consideration of sickness certification?				How often do you find it problematic to handle sickness certification?				How problematic do you generally find it to handle sickness certification of patients?			
	>10 times a week	6–10 times a week	1–5 times a week	< once a week	>5 times a week	1–5 times a week	About once a month	Less or never	Very	Fairly	Some-what	Not at all
All	14.1	19.7	47.4	18.8	4.1	27.6	37.6	30.8	7.2	31.3	45.4	16.1
*Sex*												
Women	12.2	18.8	49.8	19.2	3.3	27.0	39.0	30.7	6.7	33.1	45.7	14.5
Men	15.9	20.6	45.1	18.5	4.8	28.2	36.2	30.9	7.6	29.6	45.2	17.6
*Age (years)*												
24–39	12.9	19.8	50.6	16.7	3.1	27.5	42.2	27.2	6.2	35.4	48.7	9.7
40–54	14.9	19.9	47.7	17.4	4.4	27.5	37.0	31.1	7.4	31.3	45.0	16.3
55–67	14.3	19.4	44.1	22.2	4.8	27.8	33.7	33.7	7.9	27.4	42.8	21.9
*Specialist*												
Yes	15.1	19.9	45.1	19.9	4.5	27.0	35.5	33.1	7.4	29.1	44.3	19.2
No	11.6	19.1	52.8	16.4	3.2	29.0	42.4	25.4	6.6	36.4	48.2	8.8

### Assessment of function, work capacity, and need for sick leave

More than half of the physicians perceived it as very or fairly problematic to assess patients’ work capacity (57.8 %) and especially so if the patient was unemployed (64.4 %) (Table 3). More than half (55.7 %) also found it very or fairly problematic to provide a long-term prognosis about patients’ future work capacity. For all the studied questions, physicians in PHC had the highest ORs (range: 2.19-3.44) for finding the respective issue problematic (Table 4 and 5), compared to the reference group; internal medicine. The ORs were also higher for physicians working in rheumatology (OR range: 1.49-1.90), in psychiatry (OR range: 1.21-2.14), and in neurology (OR range: 1.34-1.84 regarding three of the items). However, for most clinical settings the ORs did not differ from the reference group or were lower. Surgery, infection, as well as ‘other clinics’ were the three clinical settings where the physicians were least likely to perceive the included issues as problematic, followed by oncology and occupational health service (Tables 4 and 5).

**Table 3:** Proportion of physicians in relation to how problematic they found different sickness certification assessments. (*n* = 12,933)

How problematic do you generally find it to…	Very	Fairly	Some-what	Not at all	Not applicable
… assess whether a patient’s functioning is reduced?	13.2	32.8	38.6	15.4	-
… assess whether the reduced functioning is due to disease/injury?	9.4	27.7	41.9	21.0	-
… assess the degree to which the reduced functioning limits a patient’s work capacity?	20.8	37.0	32.1	10.1	-
… assess the degree to which the reduced functioning limits a patient’s work capacity among those without an employment?	29.5	34.9	24.8	10.8	-
… make a long-term prognosis about the future work capacity of patients on sick leave?	21.0	34.7	30.2	14.1	-
… assess the optimum duration and degree of sickness absence?	13.2	35.3	39.5	12.1	-
… handle short-term sickness certifications					
(<15 days)?	1.6	4.6	28.2	65.5	-
… handle sickness certifications (15–90 days)?	8.8	26.9	38.6	21.1	4.7
… handle long-term sickness certifications					
(91–180 days)?	23.3	28.6	19.5	11.2	17.4
… handle very long-term sickness certifications (>180 days)?	29.9	20.9	14.1	8.6	26.6

**Table 4:** Percentages and odds ratios (OR) with 95 % confidence intervals (CI) for physicians experiencing five sickness certification assessment tasks as very or fairly problematic. Stratified by clinical settings, using physicians working in internal medicine (*n* = 1749) as reference group. The ORs were adjusted for continuous age

	Found it very or fairly problematic to …									
	… assess whether a patient’s functioning is reduced?		… assess whether the reduced functioning is due to disease/injury?		… assess the degree to which the reduced functioning limits a patient’s work capacity?		… make a long-term prognosis about the future work capacity of patients on sick leave?		… assess the optimum duration and degree of sickness absence?	
	%	OR (95 % CI)	%	OR (95 % CI)	%	OR (95 % CI)	%	OR (95 % CI)	%	OR (95 % CI)
Internal medicine	44.4	1	33.9	1	54.3	1	53.9	1	49.3	1
Orthopaedics	30.7	0.59 (0.49–0.70)	21.8	0.58 (0.48–0.70)	52.3	1.01 (0.85–1.19)	54.6	1.11 (0.94–1.32)	43.2	0.84 (0.71–1.00)
Primary healthcare	67.5	3.05 (2.71–3.44)	57.1	3.01 (2.66–3.40)	76.9	3.44 (3.03–3.89)	73.2	2.75 (2.44–3.11)	64.3	2.19 (1.94–2.46)
Occupational health service	25.2	0.66 (0.50–0.86)	20.4	0.77 (0.58–1.03)	31.2	0.65 (0.50–0.84)	35.0	0.73 (0.57–0.94)	26.6	0.61 (0.46–0.79)
Rehabilitation	24.0	0.46 (0.30–0.71)	17.4	0.47 (0.29–0.77)	35.2	0.55 (0.37–0.81)	58.4	1.45 (1.00–2.11)	30.4	0.53 (0.36–0.79)
Pain management	41.1	1.25 (0.72–2.16)	35.7	1.53 (0.87–2.68)	53.6	1.49 (0.87–2.57)	57.4	1.67 (0.96–2.91)	45.5	1.27 (0.74–2.20)
Oncology	26.7	0.49 (0.38–0.64)	19.8	0.51 (0.39–0.69)	44.1	0.72 (0.57–0.92)	48.7	0.88 (0.69–1.11)	29.2	0.45 (0.35–0.58)
Psychiatry	49.4	1.42 (1.21–1.67)	41.2	1.58 (1.33–1.86)	59.0	1.46 (1.24–1.72)	67.9	2.14 (1.81–2.54)	50.0	1.21 (1.03–1.42)
Rheumatology	50.0	1.49 (1.09–2.04)	42.9	1.73 (1.26–2.39)	64.4	1.90 (1.37–2.64)	62.8	1.74 (1.26–2.40)	54.7	1.51 (1.10–2.07)
Neurology	46.4	1.12 (0.85–1.46)	31.8	0.93 (0.69–1.24)	67.5	1.84 (1.38–2.45)	60.2	1.34 (1.02–1.76)	54.7	1.28 (0.97–1.68)
Surgery	27.5	0.47 (0.40–0.55)	20.7	0.51 (0.43–0.60)	41.8	0.60 (0.52–0.70)	38.3	0.53 (0.45–0.61)	34.4	0.53 (0.46–0.62)
Gynaecology	34.4	0.72 (0.61–0.86)	26.7	0.78 (0.64–0.93)	49.4	0.92 (0.78–1.09)	29.2	0.38 (0.32–0.45)	37.1	0.67 (0.56–0.79)
Infectious diseases	33.3	0.62 (0.48–0.80)	24.7	0.64 (0.48–0.84)	47.9	0.77 (0.60–0.98)	38.3	0.52 (0.41–0.67)	38.6	0.64 (0.50–0.82)
Dermatology	27.9	0.55 (0.39–0.79)	24.4	0.72 (0.50–1.05)	42.9	0.75 (0.54–1.04)	44.6	0.80 (0.58–1.10)	42.9	0.91 (0.65–1.25)
Others	29.6	0.56 (0.47–0.66)	22.0	0.59 (0.49–0.70)	37.8	0.55 (0.46–0.64)	36.7	0.53 (0.45–0.62)	32.8	0.53 (0.45–0.63)
Administration	42.6	1.25 (0.74–2.11)	32.3	1.23 (0.71–2.13)	52.5	1.34 (0.79–2.25)	43.5	0.89 (0.53–1.50)	40.3	0.96 (0.57–1.62)

**Table 5:** Percentages and odds ratios (OR) with 95 % confidence intervals (CI) for physicians experiencing sickness certifications of different durations as very or fairly problematic. Stratified by clinical settings using physicians working in internal medicine (*n* = 1749) as reference group. The ORs were adjusted for continuous age

	Found it very or fairly problematic to…									
	… assess the degree to which the reduced functioning limits unemployed patients’ work capacity		… handle short-term sickness certifications (<15 days)?		… handle sickness certifications (15–90 days)?		… handle long-term sickness certifications (91–180 days)?		… handle very long-term sickness certifications (>180 days)?	
	%	OR (95 % CI)	%	OR (95 % CI)	%	OR (95 % CI)	%	OR (95 % CI)	%	OR (95 % CI)
Internal medicine	63.4	1	6.1	1	32.5	1	48.2	1	44.7	1
Orthopaedics	63.2	1.07 (0.90–1.28)	9.7	1.65 (1.21–2.24)	26.2	0.77 (0.64–0.92)	55.5	1.43 (1.21–1.69)	57.1	1.72 (1.45–2.03)
Primary health care	79.1	2.59 (2.28–2.95)	5.8	0.94 (0.74–1.19)	54.5	2.70 (2.40–3.05)	76.9	4.03 (3.57–4.56)	76.4	4.32 (3.83–4.88)
Occupational health service	42.8	0.70 (0.54–0.90)	1.8	0.28 (0.12–0.66)	13.1	0.41 (0.29–0.57)	25.9	0.53 (0.40–0.69)	35.9	0.87 (0.68–1.12)
Rehabilitation	50.4	0.70 (0.48–1.01)	1.7	0.26 (0.06–1.08)	15.2	0.41 (0.25–0.67)	25.6	0.42 (0.27–0.63)	30.4	0.59 (0.40–0.87)
Pain management	55.4	1.05 (0.61–1.80)	12.5	2.17 (0.90–5.24)	23.6	0.79 (0.42–1.48)	30.4	0.61 (0.34–1.09)	35.7	0.82 (0.47–1.44)
Oncology	51.6	0.66 (0.52–0.84)	4.6	0.74 (0.43–1.30)	10.7	0.26 (0.18–0.37)	24.6	0.37 (0.28–0.48)	34.6	0.68 (0.53–0.87)
Psychiatry	67.2	1.40 (1.18–1.66)	8.7	1.46 (1.08–1.98)	32.4	1.08 (0.91–1.28)	52.0	1.30 (1.11–1.53)	55.4	1.66 (1.41–1.95)
Rheumatology	72.6	1.87 (1.32–2.64)	6.7	1.10 (0.59–2.04)	40.8	1.58 (1.15–2.16)	57.8	1.68 (1.23–2.30)	65.0	2.52 (1.82–3.47)
Neurology	72.8	1.61 (1.19–2.18)	7.4	1.23 (0.73–2.07)	31.7	0.98 (0.74–1.31)	58.1	1.53 (1.16–2.01)	55.9	1.59 (1.21–2.08)
Surgery	49.4	0.56 (0.48–0.65)	5.7	0.93 (0.68–1.26)	23.6	0.65 (0.55–0.76)	32.2	0.51 (0.44–0.59)	27.3	0.46 (0.40–0.54)
Gynaecology	58.4	0.90 (0.76–1.07)	6.9	1.13 (0.81–1.58)	27.9	0.85 (0.71–1.02)	28.3	0.45 (0.38–0.54)	18.0	0.28 (0.23–0.35)
Infectious diseases	54.3	0.68 (0.53–0.87)	2.2	0.35 (0.16–0.76)	31.0	0.94 (0.72–1.22)	40.4	0.73 (0.57–0.93)	31.1	0.56 (0.43–0.72)
Dermatology	52.4	0.74 (0.54–1.02)	7.3	1.21 (0.65–2.25)	32.7	1.10 (0.79–1.55)	37.3	0.71 (0.51–0.99)	34.5	0.70 (0.50–0.98)
Others	45.1	0.50 (0.43–0.59)	6.3	1.04 (0.75–1.44)	22.1	0.61 (0.51–0.73)	27.6	0.43 (0.36–0.51)	26.4	0.46 (0.39–0.54)
Administration	59.7	1.18 (0.70–2.00)	0.0	0 (0–0)	16.4	0.48 (0.24–0.96)	36.1	0.76 (0.45–1.30)	37.7	0.87 (0.51–1.48)

### Handling of sick-leave spells of different durations

Only a minority (6 %) found it very or fairly problematic to handle short-term sick-leave spells (<14 days). Among the physicians who handled sick-leave spells exceeding 90 days, 63 % found it very or fairly problematic to handle spells which had lasted 91–180 days, and 69 % answered the same regarding spells lasting >180 days (Table 3). Physicians in PHC had the highest ORs (range: 2.70-4.32) for finding it problematic to handle sick-leave spells with duration of at least 15 days (Table 5). These ORs were also higher among physicians working in rheumatology (range: 1.58-2.52), compared with internal medicine. Regarding sick-leave spells >90 days, the ORs were also higher for physicians working in psychiatry (1.30 and 1.66) or neurology (1.53 and 1.59). Among physicians working in orthopaedics or psychiatry, the ORs were higher (1.65 and 1.46, respectively) compared with in internal medicine, for experiencing handling of sick-leave spells of short duration (<14 days) as very or fairly problematic.

## Discussion

This large study explored how problematic physicians in different clinical settings experienced sickness certification tasks in general and related to the specific issues regarding assessment of function, work capacity, and need for sick leave as well as handling sick-leave spells of different durations – that is, essential tasks in sickness certification consultations. In summary, about a third (31.7 %) of the physicians found sickness certification consultations problematic at least once a week and found them very or fairly problematic to handle (39 %). Furthermore, more than half found it problematic to assess patients’ work capacity (57.8 %) and to make a long-term prognosis about future work capacity of patients (55.7 %). Almost half of the physicians perceived it as problematic to assess the optimum duration and degree of sick leave (48.5 %) and to assess the patients’ function (46.0 %). There were large differences between clinical settings regarding these issues. The physicians in primary health care (PHC) were by far most likely to perceive the studied issues as problematic.

### Strength and limitations

A strength of the study is that all, not a sample, physicians living and working in Sweden in a clinical setting where the physicians previously were shown to have sickness certification consultations, were included. Other strengths are the very large number of participants, making analyses of subgroups possible, the comprehensive and detailed types of survey questions, based on physicians' own experiences, and that the questionnaire had been tested and found valid in previous studies. Also, the relatively high response rate (58 %) and that the design permits analyses of bias in the drop out are strengths. Nevertheless, there was an attrition rate of 42 %, and we have no way of knowing how these physicians would have answered the different questions studied here. One reason for lower response rate among the younger physicians in this study might be changes of resident addresses due to having internship and in-residency positions at many different locations. Also, several physicians reported getting several other surveys those weeks, leading to less interest in responding.

An important limitation to studies based on survey data is that the participants might have interpreted the questions in different ways. The questions were developed in cooperation with clinicians and other researchers, and open comments to previous surveys were used in the further development of this one in order to limit uncertainties and to strengthen robustness in the definitions and the wording of the questions and to be able to assure a trustworthy interpretation of the participants’ responses.

When interpreting the results from the logistic regressions, it is important to have in mind that there were large differences in group sizes between the studied clinical settings. For example, physicians working in pain management represent a small group, meaning that the confidence intervals for this group were wide, which in turn meant that the corresponding ORs often did not reach statistical significance, while the figures for physicians in PHC, who constituted a large group, always did so.

### Sickness certification tasks in general

Some of the results from our study can be compared to those from a corresponding survey in 2008 to the physicians in Sweden [4]. In 2008 a higher proportion had sickness certification consultations more than five times a week compared with 2012 (40.3 % in 2008 compared to 33.8 % in 2012) which can be related to that a somewhat higher proportion experienced sickness certification consultations as problematic at least once a week (34.3 % in 2008 compared to 31.7 % in 2012) [4]. Only among physicians working in pain management clinics were these proportions higher in 2012.

There is no clear association between having sickness certification consultations more often and experiencing them as problematic regarding different clinical settings (Figure 1). Although only 32 % of the PHC physicians had such consultations >5 times/week they constituted the highest proportion finding them problematic. The same pattern was found for rheumatology. On the contrary, physicians in oncology had sickness certification consultations more frequently, but did not report this as problematic to any large extent, compared to e.g., those in PHC, pain management, and psychiatry. This is in line with the patterns found in the corresponding survey in 2008 [4] and underlines the robustness in the findings and the need for knowledge about factors that, in different clinical settings, influence how problematic sickness certification is experienced and factors that can support physicians in this work.

### Sickness certification tasks related to assessment of function, work capacity, and need for sick leave

The proportions who reported it problematic to assess the different insurance medicine issues were slightly (1–4 percent units) lower in our study, compared with those from the 2008 survey [4]. This can be due to fewer sickness certification cases and/or better training and organizational support for handling of these tasks. Our finding that a higher proportion of the physicians in PHC compared with physicians in other types of clinical settings experienced tasks involving assessments related to these issues as problematic are in line with previous studies from Sweden [4, 35–39]. There might be many and multifaceted reasons for why PHC physicians experience sickness certification as more problematic. It could e.g., be related to that their patients can present basically all types of symptoms, diseases, injuries, and complex psychosocial situations. This makes their work both difficult and challenging. The often very long-lasting contacts might increase the physician’s feeling of personal connection and loyalty with the patients [40, 41], which could contribute to finding it difficult to handle the two roles of being the patients’ treating physician as well as the medical expert providing other authorities with assessments [8, 18, 42].

In a randomized controlled study from Norway, it was shown that physicians in PHC were able to assess functional ability of patients in a standardized way after attending a one-day workshop to learn a method for structured functional assessments [43]. As physicians in general receive only minor training in sickness certification [14, 16], more educational efforts such as the described Norwegian method possibly could improve the physicians’ professional competence in the area and the quality of related assessments.

That physicians working in oncology and occupational health service were less likely to experience these issues as problematic is in line with the previous survey [44, 45]. Physicians working in gynaecology have been shown to find it especially problematic to handle situations when not agreeing with the patient about need for sick leave [8, 46], and more than half (52 % in 2004 and 55 % in 2008) in the former surveys also reported that they perceived it problematic to assess work capacity [46]. The corresponding proportion was, however, somewhat lower in our study (49 %), leading to the low OR for gynaecology on this variable.

### Handling of sick-leave spells of different durations

The results concerning sickness absences of different durations are in line with a previous Swiss study, where general practitioners expressed that sickness certification of absences of long duration often is problematic [47]. In Sweden, the Social Insurance Agency asks for more thorough and detailed information and assessments the longer the sick-leave spell lasts.

Two thirds (65.5 %) of the physicians reported that it was not at all problematic to handle shorter sick-leave spells. However, physicians in psychiatry and orthopaedics were more likely to perceive handling also of short spells as problematic, compared to physicians in other clinical settings. Psychiatry is a discipline where any sickness certification consultation could be a challenge, irrespective of the duration, based on difficulties establishing diagnosis and how the symptoms or disease affect the patient’s work capacity [48, 49]. In an interview study among orthopaedists, some informants did not perceive sickness certification to be part of their job [50]. That opinion could possibly have contributed to the somewhat high ORs for finding it problematic to handle sickness certifications of most durations among orthopaedists in our study.

Two thirds of the physicians (64.4 %) answered that it was problematic to assess work capacity for unemployed patients, which makes this task the one that the highest proportion of physicians rated as problematic. We have not found other studies about this – possibly partly due to that in some countries unemployed people cannot get sickness benefits.

### Implications for research and heath care management

Further studies are needed about what characterizes the clinical settings of oncology, occupational health service, surgery, and infectious diseases, e.g., regarding differing work focus, work conditions, and types of patients, for gaining knowledge about possible facilitators in physicians’ work with sickness certification. Also, issues concerning administrative support should be addressed, as many problems with handling sickness certification seem to be related to leadership and management in health care settings [51]. According to a recent study, physicians who worked in occupational health service and who had a well-established workplace policy regarding sickness certification matters were likely to find it less problematic to assess and provide a long-term prognosis of work capacity [45].

## Conclusions

About one third of the physicians found sickness certification consultations problematic at least once a week (32 %) and very or fairly problematic to handle (39 %). At a more detailed level, more than half of the physicians found it problematic to assess patients’ work capacity (59 %) and to make a long-term prognosis about patients’ future work capacity (56 %). There were large differences between clinical settings regarding experienced sickness certification problems, and physicians in PHC were by far most likely to find such tasks problematic. More knowledge is needed about the work conditions and prerequisites for optimal handling of sickness certification in different clinical settings.

## Abbreviations

CI: Confidence intervals
OR/ORs: Odds Ratio/Odds ratios
PHC: Primary health care
